# Structural and impedance spectroscopy characteristics of BaCO_3_/BaSnO_3_/SnO_2_ nanocomposite: observation of a non-monotonic relaxation behavior

**DOI:** 10.1039/c7ra12442b

**Published:** 2018-01-09

**Authors:** S. A. Salehizadeh, Hossein Mahmoudi Chenari, Mehdi Shabani, Hossein Abbastabar Ahangar, Reza Zamiri, Avito Rebelo, J. Suresh Kumar, M. P. F. Graça, J. M. F. Ferreira

**Affiliations:** Physics Department (I3N), University of Aveiro, Campus Universitario de Santiago Aveiro Portugal; Department of Physics, Faculty of Science, University of Guilan Namjoo Ave, Po Box 41335-1914 Rasht Iran; Department of Materials and Ceramic Engineering (DEMaC), University of Aveiro Campus Santiago Aveiro 3810-193 Portugal; Sustainable Developments in Civil Engineering Research Group (SDCE), Faculty of Civil Engineering (FCE), Ton Duc Thang University Ho Chi Minh City Vietnam; Department of Chemistry, Islamic Azad University Najafabad Branch Najafabad Iran; Laser Applications Research Group, Ton Duc Thang University Ho Chi Minh City Vietnam reza.zamiri@tdt.edu.vn; Faculty of Applied Sciences, Ton Duc Thang University Ho Chi Minh City Vietnam

## Abstract

A BaCO_3_/BaSnO_3_/SnO_2_ nanocomposite has been prepared using a co-precipitation method without adding any additives. The prepared sample was characterized by using X-ray diffraction (XRD), high-resolution transmission electron microscopy (HRTEM), scanning electron microscopy (SEM), Fourier-transform infrared (FT-IR) spectroscopy, energy dispersive X-ray spectroscopy (EDS) and Raman spectroscopy. Detailed studies on the dielectric and electrical behavior (dielectric constant, complex impedance Z*, ac conductivity, and relaxation mechanisms) of the nanocomposite have been performed using the nondestructive complex impedance spectroscopy technique within the temperature range 150–400 K. The dielectric constant of the sample as a function of temperature showed the typical characteristics of a relaxor. The maximum dielectric constant value was observed to depend on frequency. The non-monotonic relaxation behavior of the prepared nanocomposite was evidenced from the spectra of loss tan, tan(*δ*). The relaxation kinetics was modeled using a non-Arrhenius model.

## Abstract 2

A BaCO_3_/BaSnO_3_/SnO_2_ nanocomposite has been prepared using a co-precipitation method without adding any additives.

## Graphical abstract

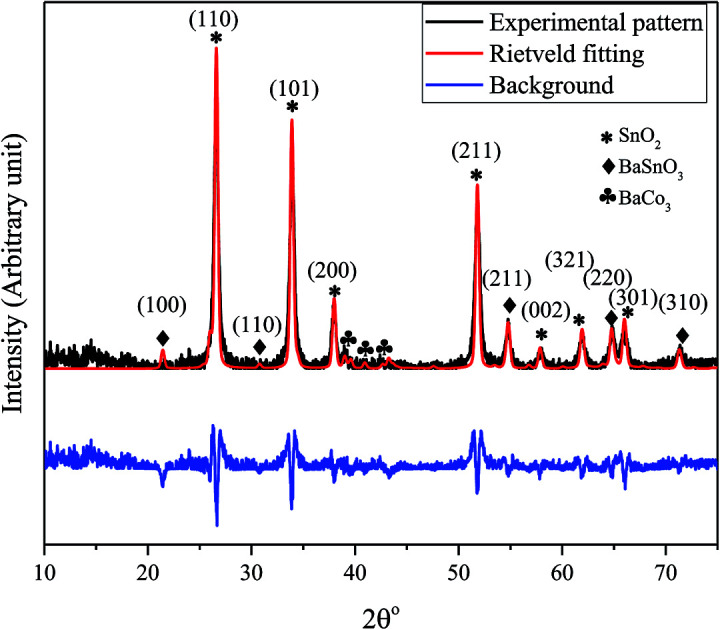

## 1. Introduction

Over the last two decades barium stannate, BaSnO_3_, from the perovskite family with the general formula of ABO_3_ (A = mono/di, B = tri/tetra/penta) has received increasing attention for humidity sensors, computer memories, pyroelectric detectors and electro-optic modulators, due to its superior optical and electrical nature.^1–4^

Barium stannate is also an important n-type semiconductor resulting from the formation of oxygen vacancies or by suitably doping at the Ba and/or Sn sites.^5,6^ However, this perovskite-like material is also interesting for other applications like ceramic capacitors due to the characteristics of its dielectric constant.^7,8^

It is complicated to synthesize pure single phase of BaSnO_3_. It was shown that the highly crystalline barium stannate could be conventionally obtained by a solid-state calcination process at *T* > 1200 °C.^9,10^ Recent studies on the kinetics and mechanism of solid state reaction clearly showed that the particle size reduction of the raw materials (BaCO_3_ and SnO_2_) to the submicrometer or even the nanometer scales results in a significant decrease of the synthesis temperature of the perovskite material. Therefore, some wet chemistry routes, such as alkoxide hydrolysis, sol–gel,^6,11,12^ hydrothermal,^13,14^ and co-precipitation^15^ methods were proposed as effective ways to shorten the reactions paths. In these methods, the main solid reactants (*e.g.* BaCO_3_ and SnO_2_) could be mixed at the nanoscale, which led to the formation of BaSnO_3_ nanopowders at a much lower temperatures of 500–900 °C.^6,11–15^ However, the large amounts of organic solvents or reacting agents required for these routes could be hardly removed from the systems. This is likely to change the electrical performance due to contrasting properties depending upon the type of forming system and the mixed solid solution.^16^ It includes phase transition (ferro–paraelectric), dielectric and alternates (increase/decrease) in resistance with temperature and frequency.^17^ The obtained modification makes this material suitable and useful for many industrial applications.

In ferroelectric materials with perovskite structure (ABO_3_) the inherent anisotropy arised from high degree of microstress and structural defects, including the random distribution of oxygen defects and dopant, make these ceramics promising for possible applications in various technologies like memory storage devices, microelectro mechanical systems, multilayer ceramic capacitors, and recently in the area of opto-electronic devices.^18^

Relaxor ferroelectrics (“relaxors” for short) have attracted significant interest due to their superior dielectric and electro mechanical properties.^19^ The most typical feature of relaxors is a strongly broadened frequency-dependent peak of the dielectric permittivity *versus* temperature, whose position shifts to lower temperatures when the frequency decreases.^19^ Several models have been proposed to explain the relaxation behavior in relaxors.^18,20,21^ A commonly proposed explanation is that it arises from the motion of polar nanoregions due to the local distortion of the structure coming from chemical inhomogeneity.^20–22^ The existence of the local nanometric clusters can be confirmed experimentally using several measurements including optic index of refraction,^23^ synchrotron-ray scattering,^24^ diffuse neutron scattering,^25^ and transmission electron microscopy.^26^ Moreover, dielectric spectroscopy measurements in broad temperature and frequency ranges are fruitful to investigate the relaxational behavior of the clusters in a relaxor.^19–21^ However, the origin of this dipolar relaxation is still open for discussion.

In the present work, the structure and morphology of the BaCO_3_/BaSnO_3_/SnO_2_ nanocomposite synthesized by precipitation method has been studied using X-ray diffraction, FT-IR, Raman spectroscopy, SEM, and TEM techniques. These studies indeed prove the predominance of the reactants BaCO_3_ and SnO_2_ in the sample, and the formation of nanoscale particles of BaSnO_3_. To have a better understanding of the electric and dielectric behaviors of the material, these properties were studied within wide ranges of frequency and temperature. The observation of the relaxational behavior is modeled and discussed using the proper theoretical model.

## 2. Experimental procedure

In a typical experiment, appropriate amounts of SnCl_4_·5H_2_O (0.01 mol) and BaCl_2_·2H_2_O (0.01 mol) were separately dissolved in deionized water under continuous stirring for 30 minutes at room temperature. A quantitative volume of aqueous NaOH solution was slowly added into the mixture solution under vigorous stirring to promote the precipitation. The precipitate was filtered and washed with deionized water for several times, and then dried at 100 °C. The white precipitate was calcined at 500 °C for 5 h to obtain BaCO_3_/BaSnO_3_/SnO_2_ nanocomposites.

The precipitate formation can be summarized by the following reactions:^27^
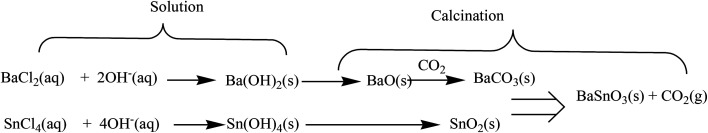


The synthesized nanoparticles were then used for experimental characterization studies. Phase identification was performed using X-ray diffractometer (XRD; X'Pert Philips Materials Research System) with Cu-Kα radiation (*λ* = 1.5406 Å) under accelerating voltage of 45 kV and current of 40 mA in the 2*θ* range from 20–80°. Powder pellets were molded into disks (thickness ≈ 2.0 mm; diameter = 7 mm). The bulk sensitive X-ray diffraction (XRD) patterns were acquired with a Philips X'Pert MPD equipment with Cu K_α_, *λ* = 1.5406 Å (40 kV). The Raman spectroscopy of the pellets was carried out in a T64000 JobinYvon SPEX spectrometer, using an Ar laser (*λ* = 532 nm) as excitation font. The spectra were obtained between 100 and 1200 cm^−1^ in a back scattering geometry.

For the electrical measurements the opposite sides of the pellet were painted with silver paste. Impedance spectroscopy measurements were performed in temperature range of 150 to 400 K, between 10^2^ Hz and 10^6^ Hz using an Agilent 4294A network analyzer in the Cp–Rp configuration.^28^

## 3. Results and discussion

**Fig. 1:**
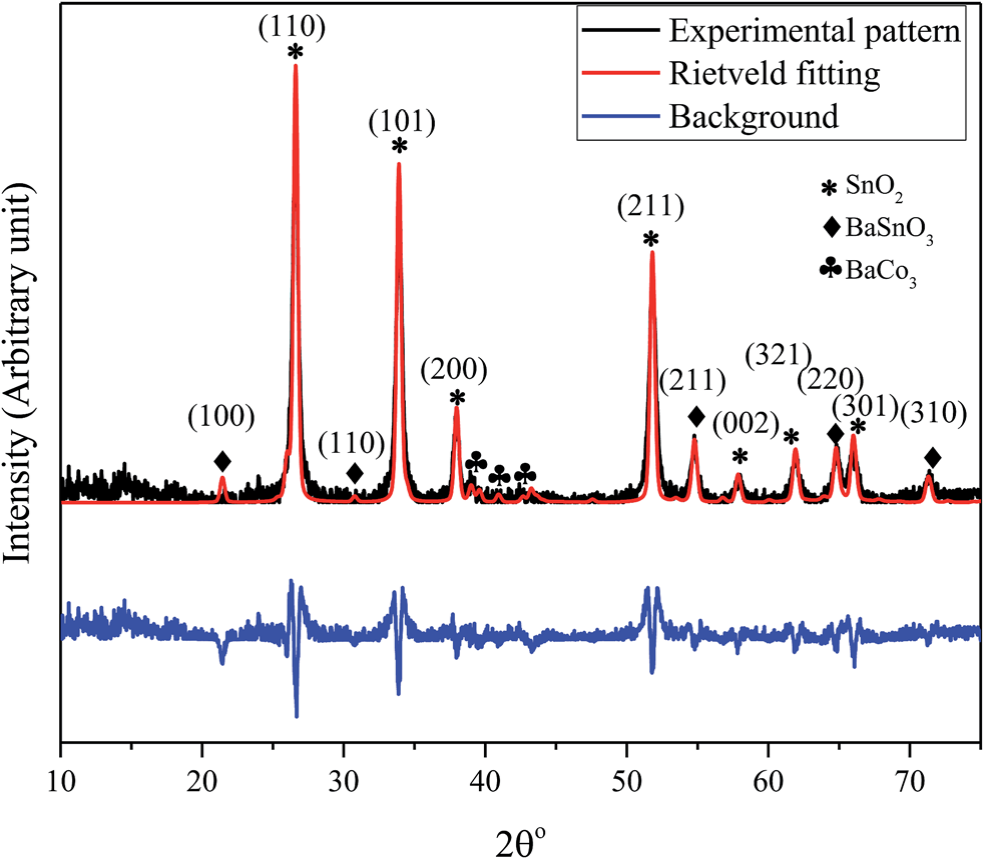
XRD pattern and the Rietveld refinement of BaCO_3_/BaSnO_3_/SnO_2_ nanocomposite powder along with the crystal plan indexation for the main phases.

The X-ray diffraction pattern of the nanocomposite powder sample calcined at 500 °C shown in Fig. 1 reveals the existence of a mixture of BaCO_3_/BaSnO_3_/SnO_2_ phases. The Bragg's peaks for the predominant SnO_2_ phase correspond to the tetragonal symmetry, consistent with the JCPDS No. 41-1455. The XRD peaks of BaCO_3_ are in agreement with the orthorhombic structure and space group of *Fd*3̄*m* (JCPDS card 44-1487). In addition, the reaction between BaCO_3_ and SnO_2_ led to the formation of small amounts of an intermediate phase, cubic BaSnO_3_ as indicated by the XRD line (110). The small content of cubic BaSnO_3_ can be attributed to the relatively low calcination temperature. Additionally, Reference Intensity Ratio (RIR) method was employed to quantitatively determine the proportion of each component within the nanocomposite. The analysis revealed that the prepared composite consists of 71% SnO_2_, 17% BaSnO_3_, with only 12% of BaCO_3_. This was further supported by an exclusive Raman study on the composition, as will be discussed below. The reflection peaks corresponding to the main phases are indicated in Fig. 1.

### 3.1. SEM/EDS and TEM analyses

**Fig. 2:**
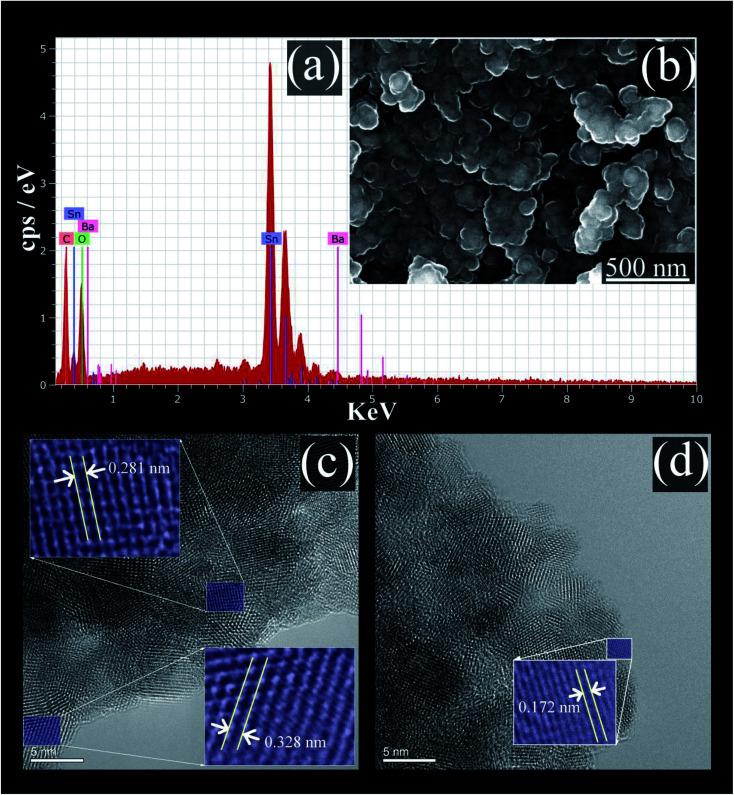
(a) The EDS spectrum; (b) SEM micrograph; (c) and (d) HRTEM images of BaCO_3_/BaSnO_3_/SnO_2_ nanocomposite.

The EDS analysis spectrum, and SEM image, of the prepared BaCO_3_/BaSnO_3_/SnO_2_ nanocomposite are shown in Fig. 2a and b, respectively.

The EDS analysis confirms that the product contains Ba, Sn, C and O without the presence of any impurity. The SEM image reveals that spherical shaped nanoparticles were initially formed and then tended to merge partially into agglomerates. In addition, the lattice plane fringes from HRTEM images were used to calculate the *d*-spacing values (Fig. 2c and d). The *d*-spacing's equal to 0.328 nm, 0.172 nm, and 0.281 nm correspond to the (110), (113), and (110) crystal planes of SnO_2_, BaCO_3_, and BaSnO_3_, respectively.^29^

### 3.2. FT-IR spectroscopy

**Fig. 3:**
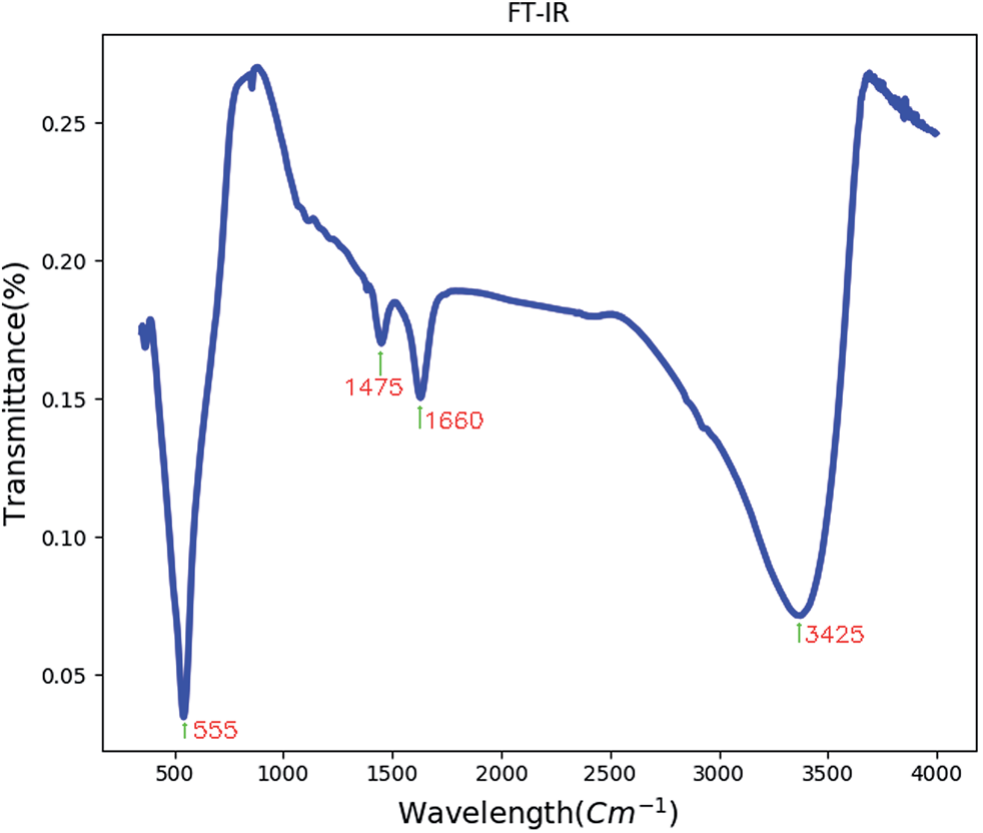
The FT-IR spectrum of BaCO_3_/BaSnO_3_/SnO_2_ nanocomposite.

Fig. 3 shows the FT-IR spectrum of BaCO_3_/BaSnO_3_/SnO_2_ nanocomposite. The peak appearing at 550 cm^−1^ corresponds to the Sn–O stretching vibration.^13^

The band centered at 1660 cm^−1^ is related to the C–O stretching vibrations and C–C stretching vibration of the BaCO_3_. Absorption peak at 1475 cm^−1^ may correspond to carbonate group in BaCO_3_. The band centered at 3425 cm^−1^ corresponds to the *ν*(OH) stretching vibration of the surface adsorbed water molecules.^30,31^

### 3.3. Raman spectroscopy

**Fig. 4:**
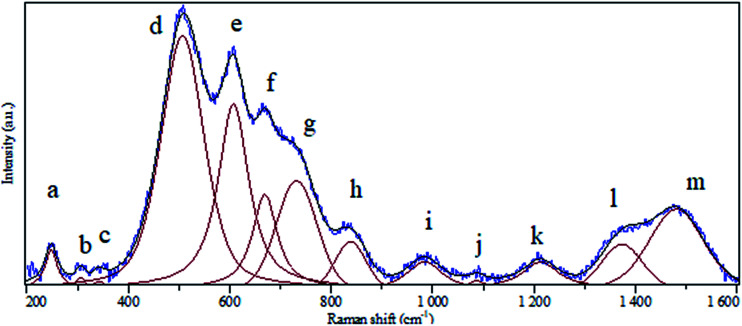
Raman spectrum of BaCO_3_/BaSnO_3_/SnO_3_ nanocomposite.

A clear batch of Raman active modes was detected for the case of BaCO_3_/BaSnO_3_/SnO_3_ nanocomposite. The deconvolution of Raman spectrum for this composition was carried out using Gaussian fitting as described in ref. 32 and 33 to identify and assign all possible vibrational modes. The peak positions labeled in alphabetic order, and the assignments of the modes could be precisely obtained, being presented in Fig. 4 and reported in Table 1, respectively. For the case of BaCO_3_, the free ion CO_3_^2−^ with *D*_3h_ symmetry exhibits four normal vibrational modes: a symmetric stretching vibration (1); an autoquantitative analysis of the of-plane bend (2); a doubly degenerate asymmetric stretch (3); and a doubly degenerate bending mode (4). The symmetries of these modes are A_1′_(R) + A_2′′_ (IR) + E′(R, IR) + E′(R, IR) occur at 1064, 879, 1415 and 680 cm^−1^, respectively. The symmetric stretching vibration is very intense in the Raman spectrum, while the asymmetric stretching is weak. The asymmetric bending, although Raman-allowed, is very weak.^3,34,35^

**Table 1:** Peak positions and assignments for the deconvoluted Raman spectrum of BaCO_3_/BaSnO_3_/SnO_3_ nanocomposite

Peak notation	Position (cm^−1^)	Assignment	
a	247	Corresponds to defect or oxygen vacancy in BaSnO_3_	
b	306	Corresponds to defect or oxygen vacancy in BaSnO_3_	
c	342	Corresponds to defect or oxygen vacancy in BaSnO_3_	
d	507	Sn–O–Sn vibration mode (B_1u_ symmetry)	
e	607	Sn–O vibration mode (A_1g_ symmetry) in SnO_2_	
f	669	Sn–O vibration mode (A_2u_ symmetry) in SnO_2_	
g	731	Sn–O vibration mode (LO symmetry) in SnO_2_	
h	838	Bending in-plane vibration in BaCO_3_	
i	984	Corresponds to defect or oxygen vacancy in BaSnO_3_	
j	1087	Bending in-plane vibration in BaCO_3_	
k	1212	Corresponds to defect or oxygen vacancy in BaSnO_3_	
l	1373	Corresponds to defect or oxygen vacancy in BaSnO_3_	
m	1480	Asymmetric stretching vibration in BaCO_3_	

Rutile tetragonal SnO_2_, containing two Sn and four O atoms in a single unit cell, belongs to the space group *D*^14^_4h_. According to the group theory, the representation of normal vibration modes at the center of the Brillouin zone is *Γ* = A_1g_ + A_2g_ + 2A_2u_ + B_1g_ + B_2g_ + 2B_1u_ + E_g_ + 4E_u_.^36^ However, we could not find all the modes regarding to SnO_2_ and BaCO_3_. In sufficient intensity arising from small polarizability of several modes could be the reason for the less number of modes observed relatively to that predicted by group theoretical calculation.^37^

It should be pointed out that there are no Raman-active modes in the ideal perovskite crystal of the centrosymmetric BaSnO_3_ with ideal cubic structure belonging to the space group *Pn*3*m* because of inversion symmetry of all-atom occupying site.^3^ Although some of modes could be assigned, there are no assignments in literature corresponding to the detected a, b, c, i, k and l modes. It is likely that these modes are induced by defects such as oxygen vacancies, which destroy the translational periodicity of the lattice BaSnO_3_.^3^ To confirm the result of RIR analysis we have calculated the relative area of the bands pertaining to each phase to qualitatively determine the abundance of each component in the composite using the following relation:

1 *R*_phase(i)_ (in %) = (area of bands assigned to phase_(i)_/total area of the spectrum) × 100

According to eqn (1), *R*_SnO_2__, *R*_BaSnO_3__ and *R*_BaCO_3__ were found to be 67%, 18% and 15%. These values are very consistent with the ones obtained from RIR analysis.

### 3.4. Impedance spectroscopy

**Fig. 5:**
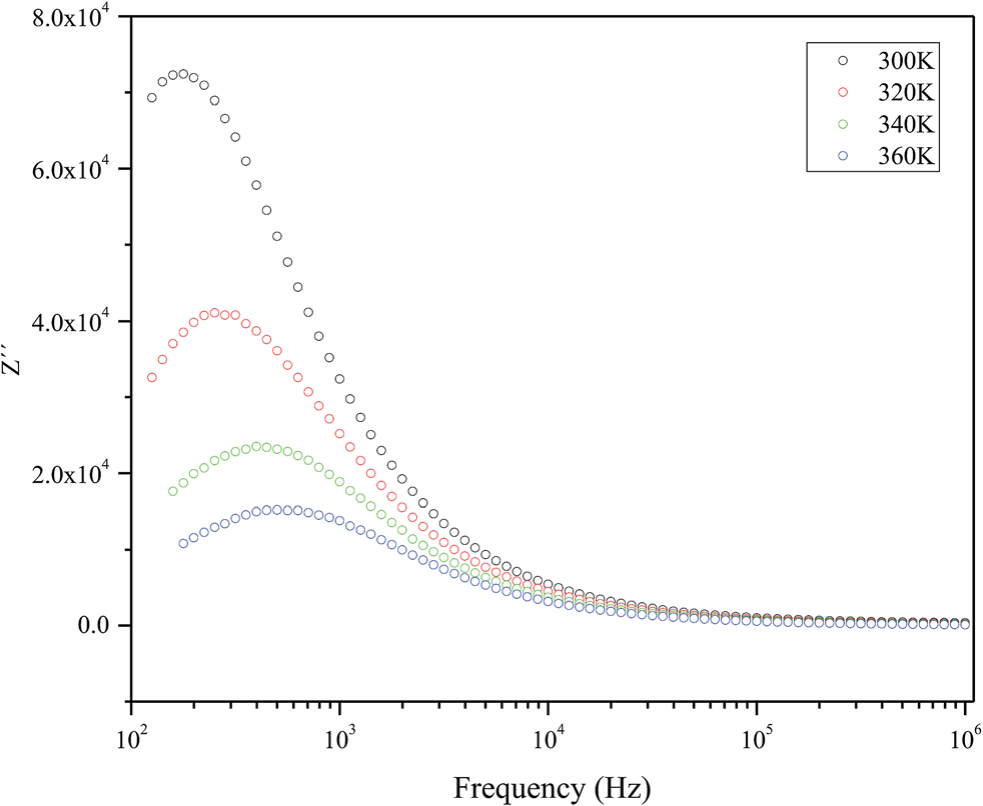
The frequency dependency of imaginary part of impedance (*Z*′) measured at selected temperatures.

Fig. 5 represents the variation of imaginary part of impedance (*Z*′) with frequency (Hz). The variation pattern is characterized by: (a) the appearance of peaks at a particular frequency at each temperature; (b) a decrease in the height of the peaks with rise in temperature; (c) significant broadening of the peaks with rise in temperature; (d) marked asymmetry in the peak pattern; and (e) merging of the spectra at higher frequencies irrespective of temperature. Broadening of the peaks with the rise in temperature suggests the presence of temperature-dependent relaxation process in the material. The asymmetric broadening of the peaks suggests the presence of electrical processes in the material with the spread of relaxation time (indicated by peak width) with two equilibrium portions. The relaxation species may possibly be electrons/immobile species at lower temperatures, and defects at a higher temperatures that may be responsible for electrical conduction in the material by hopping of electrons/oxygen ion vacancy among the available localized sites.^38,39^

**Fig. 6:**
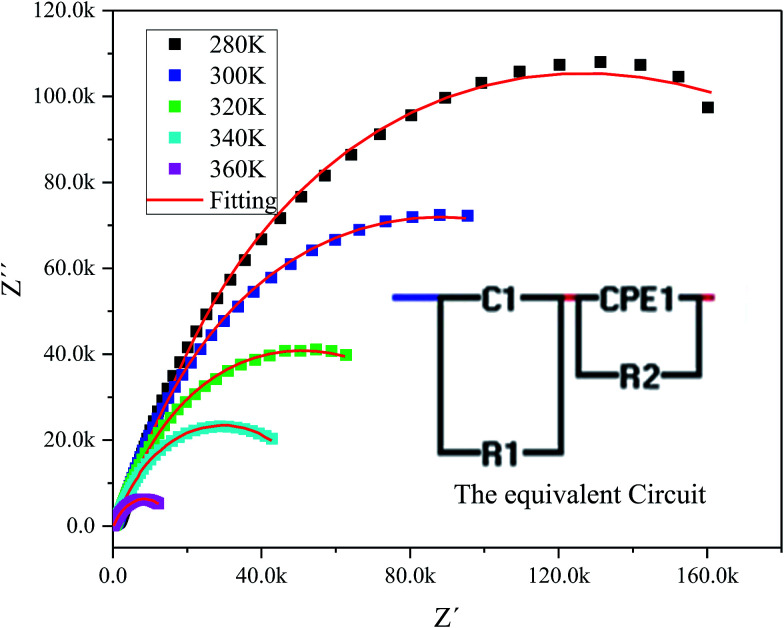
The experimental complex impedance spectra combined with the fitting curves modeled by the equivalent circuit (inset).

The complex impedance spectrum is a powerful method to study the relaxation polarization mechanism of the grain and grain boundaries in ceramics.^40^Fig. 6 shows the variation of the real part of impedance (*Z*′) with the imaginary part of impedance (*Z*′) (Nyquist plots) for sample at different temperatures (from 280 to 360 K).

The Nyquist plots feature single semicircles with radii of curvatures decreasing with temperature increasing. In order to model the physical interpretation of the distributed elements, the experimental data were fitted to an equivalent circuit combining a parallel grain resistance (*R*–*C*_1_) and constant phase element impedance (CPE) (*R*–CPE circuit) (inset of Fig. 6) by means of ZView Software. Through this equivalent circuit we could obtain the best fit to the data measured at different temperatures. The experimental spectra of the sample along with the fitting curve are shown in Fig. 6b. The CPE impedance (CPE) is given by the following relation:^41^2
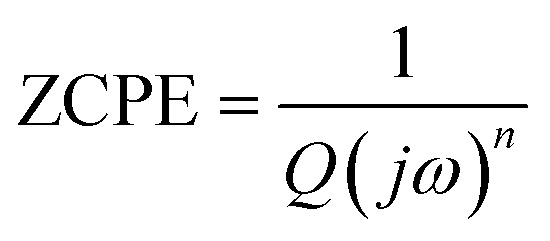
where *Q* is a proportional factor, *ω* is the angular frequency and *n* is an empirical exponent with values between 0 and 1. The parameters extracted from the fitting are summarized in Table 2.

**Table 2:** The parameters of the interior grain component (*R*_g_, *C*_g_) and the grain boundary components (*R*_gb_, CPE_gb_, *n*_gb_) obtained from the fitting to the assumed in the equivalent circuit along with *n* in eqn (4) extracted for the region III^a^

Temperature (K)	*R*_g_	*C*_g_	*R*_gb_	CPE_gb_	*n*_gb_	*n*_III_
280	1.45 × 10^5^	6.18 × 10^−9^	2.55 × 10^5^	4.08 × 10^−7^	0.53	0.31
300	8.89 × 10^4^	8.36 × 10^−9^	1.35 × 10^5^	2.45 × 10^−7^	0.60	0.33
320	4.92 × 10^4^	1.01 × 10^−8^	6.65 × 10^4^	2.31 × 10^−7^	0.63	0.35
340	2.82 × 10^4^	1.06 × 10^−8^	4.80 × 10^4^	5.25 × 10^−7^	0.57	0.4
360	6.97 × 10^3^	1.30 × 10^−8^	1.24 × 10^4^	6.99 × 10^−7^	0.58	0.5
400	—	—	—	—	—	0.37

a Both *R*_gb_ and *R*_g_ values are decreasing as temperature increases indicating a negative temperature coefficient of resistance (NTCR) behavior which can be explained by the increment of the mobility of charge carriers.^42^

Furthermore, the obtained values can be used to calculate the activation energies for grain-interior and grain boundary conductivity. The grain and grain boundary conductivity can be defined as a function of temperature following the Arrhenius law:^43^3
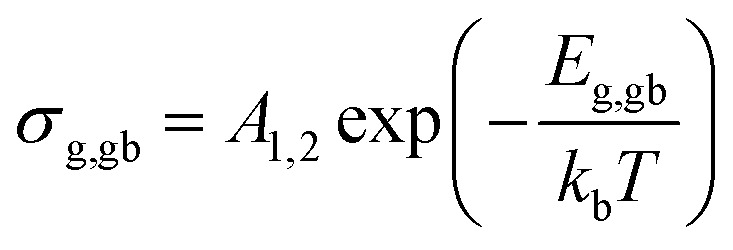
where *A*_1,2_ are pre-exponential factors, *E*_g,gb_ the activation energies of grain and grain boundaries, and *k*_b_ is the Boltzmann constant. The calculated values of *E*_g_ and *E*_gb_ were found to be 20.2 and 21.6 kJ mol^−1^, respectively. The greater contribution of grain boundaries for the activation energy attests its resistive nature. Migration of oxygen vacancies and also Ba and Sn ions are hindered due to compositional inhomogeneity (provoking electrical inhomogeneity) in the prepared composite. Therefore, *E*_gb_ increases. This behavior is similar to that reported by Markovic *et al.*^44^ showing that contacting inhomogeneities between grains in barium titanate stannate material increase *E*_gb_.

**Fig. 7:**
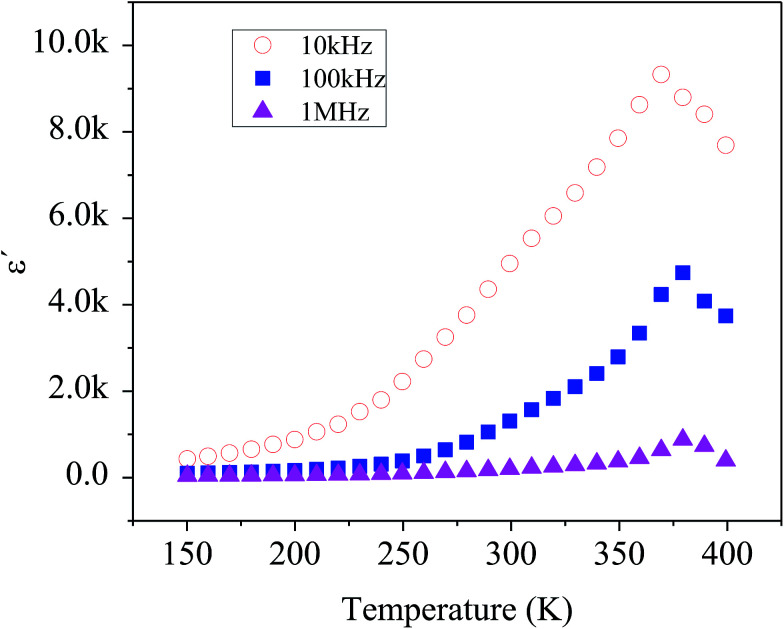
Temperature dependence of the dielectric constant measured at different frequencies.

Fig. 7 shows the temperature dependency of dielectric constant for the composite at several frequencies. Variation of dielectric constant as a function of temperature indicates the appearance of a peak at a particular temperature (*T*_max_) ranging from 370–380 K with increasing in frequency from 1 kHz to 1 MHz, attributed to the ferroelectric–paraelectric phase transition. This observation is in good agreement with the previous reports on the presence of phase transition corresponding to the ferroelectric–paraelectric in compounds with perovskite (ABO_3_) structure^8–10^ and also with ABO_4_ structure.^11–14^ This *T*_max_ value is much lower than 800 K for Te modified BaSnO_3_ and larger than 200 K for highly doped Pb–BaSnO_3_.^45^ Considering the inhomogeneity within the nanocomposite (see Raman, XRD and TEM data), it is obvious that atoms undergo a notable displacement with respect to their high-symmetry locations. Hence, there is a local dipole moment in both Ba–O and Sn–O planes which gives rise to spontaneous polarization. Moreover, compressive strain due to the heterogeneity of the structure leads to the emergence of out of phase oxygen octahedral rotation along the [110] axis resulting in an out of plane polarization along the [110] direction. Thus, the zone-center polar lattice distortion completely accounting for the symmetry lost and also oxygen octahedral rotation and tilting along [110] direction is responsible for the paraelectric–ferroelectric transition.^46,47^

**Fig. 8:**
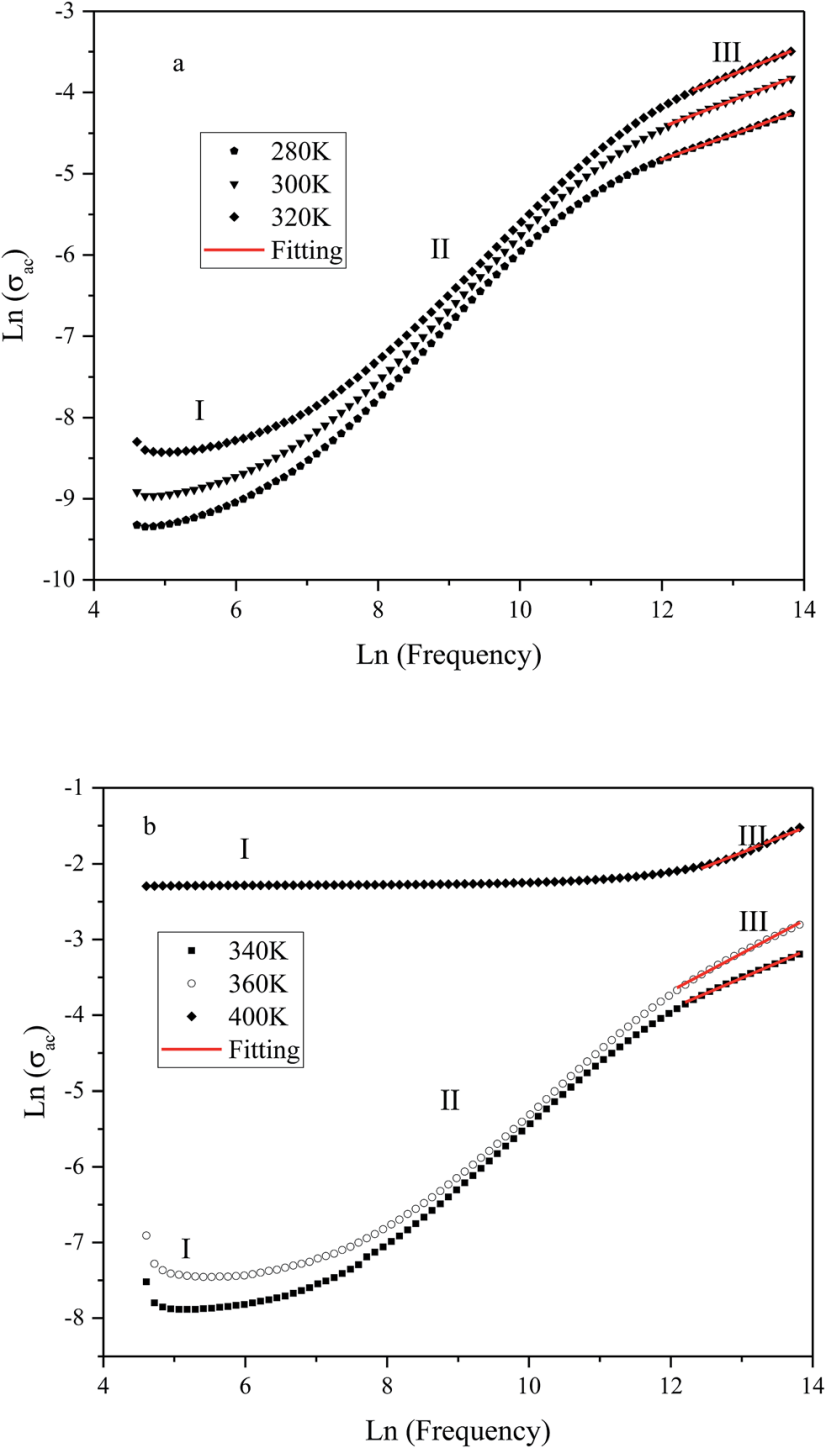
Variation of ac conductivity (*σ*_ac_) as function of frequency at different temperatures (K): (a) 280, 300 and 320; (b): 340, 360 and 400.

The variation of ac conductivity (*σ*_ac_) of the composition as a function of frequency at different temperatures is shown in Fig. 8a and b. It is observed that at lower temperatures three distinct regions are visible in the conductivity spectra of the sample: in region I, conductivity curve is nearly frequency independent; in region II, conductivity increases almost proportionally with increasing frequency; whereas in region III the increasing trend of conductivity continues but at gradually slower rates due to a different type of response of bound charge carriers at higher frequencies in comparison with region II. The levels of low-frequency conductivity plateau increase with increasing temperature. These results indicate the thermally activated process of electrical conduction in close agreement with the observations from impedance spectrum results. Dispersion with different slope in the intermediate region can be due to multiwell potential barriers coming from non-homogeneity and random distribution of defects.

These results suggest that electrical conduction in the material takes place *via* hopping mechanism governed by the Jonscher's universal power law:^1^

4 *σ*_ac_ = *σ*_dc_ + *Aω*^*n*^

where *A* is a thermally activated constant depending upon temperature. We have tried to fit the region III of the spectra to eqn (4). The obtained values for *n* varies between 0.31 and 0.50 depending upon temperature as you can see in Table 2. The exponent *n* represents the degree of interaction between the mobile ions. *n* = 1 represents non-interacting Debye system and with decreasing *n*, interaction between mobile ions and lattice is expected to increase.^48^ In the ferroelectric region, the *n* values were found to be both temperature and frequency dependent, indicating that the conductivity is due to phonon-assisted tunneling between defect states.^49^ However, at temperatures higher than *T*_max_, the *n* values decrease. This is due to an increase in randomness in the system.^48^ It is believed that for temperatures above the ferroelectric–paraelectric transition, the oxygen vacancies can interact strongly with the lattice and distort the Ba^2+^ and Sn^4+^ ions.

**Fig. 9:**
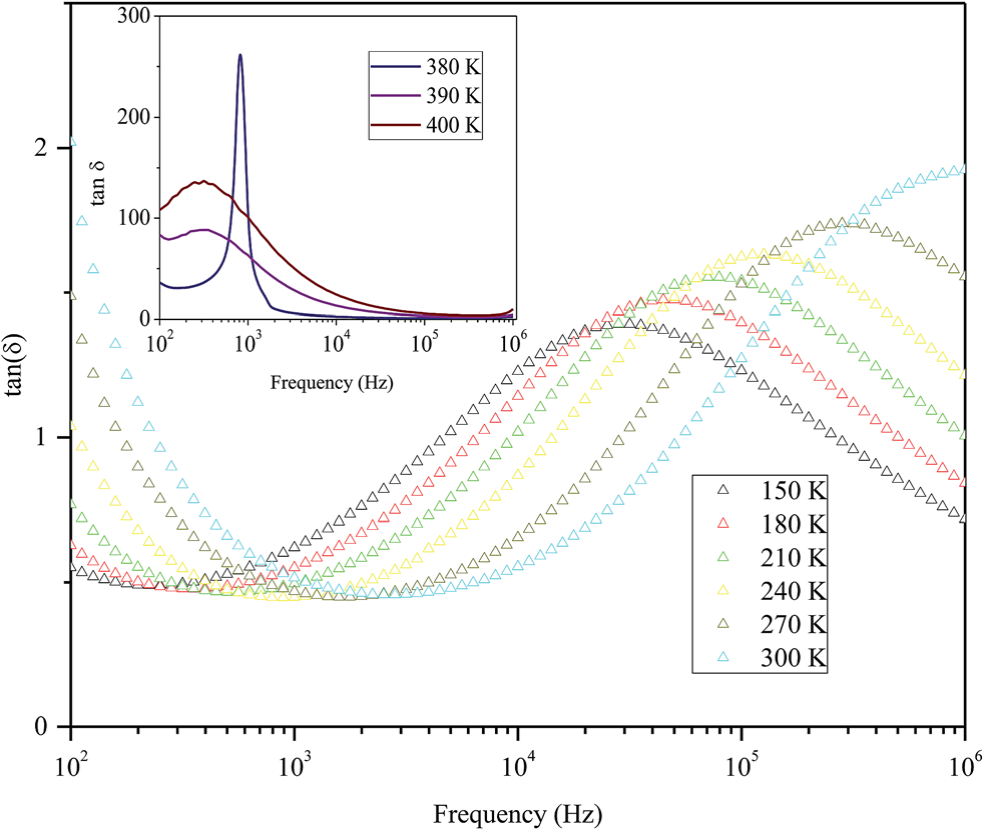
Variation of tangent loss as a function of frequency at selected temperatures below and above phase transition of the nanocomposite.

**Fig. 10:**
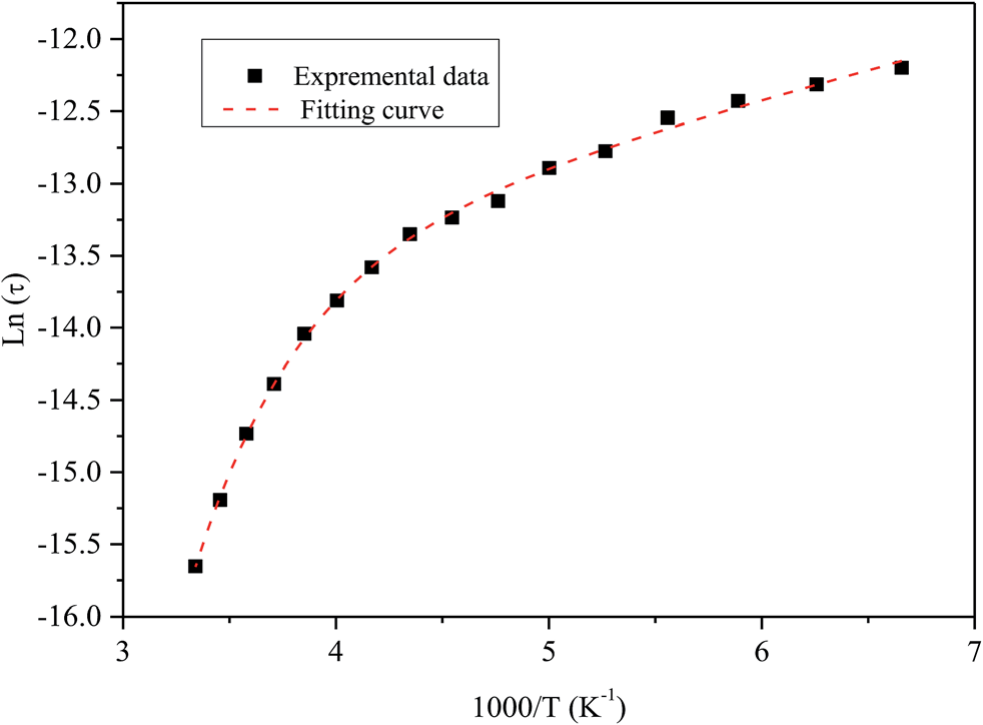
Temperature dependence of the tan(*δ*) relaxation time of the nanocomposite. Symbol (black) represents experimental data. The dashed line (red) corresponds to the best fit according to the eqn (5).

Fig. 9 shows the variation of tangent loss as a function of frequency at selected temperature (below and above the phase transition). It can be seen that tan(*δ*) increases gradually when the frequency values rise, reaching a maximum, and then decreasing, indicating the presence of a relaxation phenomenon. The tan(*δ*) peak shifts towards higher frequencies as temperature increases in the ferroelectric region (the temperatures below the phase transition). Inversely, for the paraelectric region, above the phase transitions, the peak shifts backwardly to the lower frequencies. It is worth noting that no relaxation was observed in temperature range of 310–370 K. The temperature dependency of the tan(*δ*) relaxation time is presented in Fig. 10. Quantitative examination of the relaxation time revealed that it follows a non-Arrhenius behavior in the ferroelectric region. The nonmonotonic behavior of relaxation kinetics for our nanocomposite can be discussed as a complex phenomenon, having its origin in two different processes.^50^ The first process is the tilt of oxygen octahedron, which changes the orientation of the elementary dipole moments, associated with the perovskite unit cell, and can be presented by an Arrhenius term with activation energy of *E*_a_. The second process is the jump of Ba and Sn ions between the several local minima positions created due to the heterogeneity of the composition. For the latter process, the heterogeneous structure creates “defects” with multiwall potentials in the unit cell structure and hinder tilting of the oxygen octahedrons. Hence, the increasing number of such “defects” with an increase in temperature slows down the relaxation process and can be described by the exponential term with the activation energy of *E*_b_. This argument is in good similarity with that for evaluation of the non-monotonic relaxation kinetics observed in the copper-doped KTa_1−*x*_Nb_*x*_O_3_ ferroelectric perovskite.^50^ Thus, were present a non-monotonic temperature dependence of the relaxation time since it is related to two processes of a different nature, in the form of:^51^5
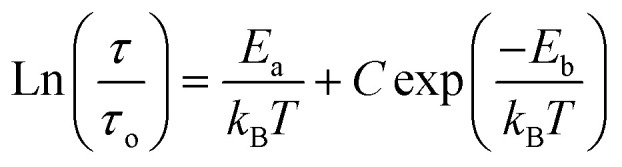
where *k*_B_ is the Boltzmann constant and *C* is a constant related to the defect concentration. The fitting curve presented in Fig. 10 shows that the model is in good agreement with the experimental data. The fitted values of *E*_a_ and *E*_b_ are within the ranges of 3.34 ± 0.01 kJ mol^−1^ and 16.43 ± 0.01 kJ mol^−1^, respectively. The activation energy *E*_b_ is five times larger than *E*_a_. The necessary energy to overcome the high concentration of defects is higher than the energy needed for tilting the oxygen octahedron, resulting from heterogeneities in the prepared nanocomposite.

## 4. Conclusion

A BaCO_3_/BaSnO_3_/SnO_2_ heterogeneous nanocomposite was prepared *via* precipitation–calcination method. The phase identification, structural and morphological evolutions, was extensively investigated using XRD, FT-IR, Raman, SEM and TEM analyses.

The complex impedance plots revealed the existence of grain and grain boundary contributions. *Z′* showed distributed relaxation phenomena at higher temperatures, and was found to be temperature dependent. The temperature dependence of and tangent loss is characterized by the appearance of a peak ranging from 370–380 K, depending on the frequency. This phenomenon is related to the ferroelectric–paraelectric phase transition, due to the tiling of the oxygen octahedral and the zone-center polar lattice distortion. The larger contribution of the grain boundary for conduction was evaluated considering the high concentration of oxygen vacancies and other compositional heterogeneities.

In the paraelectric state, electrical conduction was again associated with the oxygen vacancies. The non-monotonic dependence of the relaxation time of tan(*δ*) on temperature was explained using a non-Arrhenius model.

## Conflicts of interest

There are no conflicts to declare.

## Supplementary Material
